# Laparoscopic Boari flap for treatment of benign midureter stricture

**DOI:** 10.1590/S1677-5538.IBJU.2018.0423

**Published:** 2020-02-20

**Authors:** Willian Eduardo Ito, Andre Fernando Tannouri Garbin, Marco Aurelio de Freitas Rodrigues, Silvio Henrique Maia de Almeida, Horacio A. Moreira

**Affiliations:** 1 Universidade Estadual de Londrina Disciplina de Urologia LondrinaPR Brasil Disciplina de Urologia, Universidade Estadual de Londrina, Centro de Ciências da Saúde, Londrina, PR, Brasil

## Abstract

**Introduction:**

Laparoscopic ureteral reconstructive surgery represents a real challenge for most of the urologists as it requires advanced skills. Impacted stones (>2 months) and endoscopic procedures are known major risk factors for ureteral strictures. Boari flap is a good alternative, due to the high recurrence of kidney stone disease, as it preserves the feasibility of ureteroscopy.

**Material and Methods:**

We present a case of a 21-year-old female patient complaining of dull pain in the left flank, associated with vomiting and high-grade fever (39 degrees Celsius), for three days. Computed abdominal tomography demonstrated a 16mm ureteral stone in the left mid-ureter. Besides intravenous antibiotics, we installed a retrograde pigtail ureteral stent, a difficult procedure, due to extended length stenosis (retrograde pyelography, ~6cm). Two weeks after clinical improvement, we conducted a laparoscopic transperitoneal Boari flap for definitive treatment.

**Results:**

Surgery had a duration of 169 minutes and 100mL of bleeding. The calculus was retrieved along with the fibrotic ureteral tissue. Psoas-Hitch was not needed and end-to-end flap-ureteral anastomosis was done using polyglactin 4.0 continuous sutures. Intraoperatively we had no significant issues. The patient was discharged three days post-operatively. Foley catheter was maintained for 14 days, and it was withdrawn after a cystography, ureteral stent was left for four weeks. Six weeks after the procedure, a urography was done, which showed a normal full bladder capacity and optimal drainage of the left kidney.

**Conclusion:**

Laparoscopic Boari flap is feasible, resolutive and a safe minimally invasive technique for the treatment of mid-ureteral strictures.

